# Genetic discrimination and life insurance: a systematic review of the evidence

**DOI:** 10.1186/1741-7015-11-25

**Published:** 2013-01-31

**Authors:** Yann Joly, Ida Ngueng Feze, Jacques Simard

**Affiliations:** 1 Department of Human Genetics, Faculty of Medicine, McGill University, 740 Dr Penfield Avenue, Suite 5200, Montreal, H3A 1A5 Canada; 2 Department of Molecular Medicine, Faculty of Medicine, Laval University, 2705 Boulevard Laurier, Quebec City, G1V 4G2 Canada

**Keywords:** Evidence, genetic discrimination, genetic exceptionalism, GINA, life insurance, personalized medicine, stigmatization, systematic review

## Abstract

**Background:**

Since the late 1980s, genetic discrimination has remained one of the major concerns associated with genetic research and clinical genetics. Europe has adopted a plethora of laws and policies, both at the regional and national levels, to prevent insurers from having access to genetic information for underwriting. Legislators from the United States and the United Kingdom have also felt compelled to adopt protective measures specifically addressing genetics and insurance. But does the available evidence really confirm the popular apprehension about genetic discrimination and the subsequent genetic exceptionalism?

**Methods:**

This paper presents the results of a systematic, critical review of over 20 years of genetic discrimination studies in the context of life insurance.

**Results:**

The available data clearly document the existence of individual cases of genetic discrimination. The significance of this initial finding is, however, greatly diminished by four observations. First, the methodology used in most of the studies is not sufficiently robust to clearly establish either the prevalence or the impact of discriminatory practices. Second, the current body of evidence was mostly developed around a small number of 'classic' genetic conditions. Third, the heterogeneity and small scope of most of the studies prevents formal statistical analysis of the aggregate results. Fourth, the small number of reported genetic discrimination cases in some studies could indicate that these incidents took place due to occasional errors, rather than the voluntary or planned choice, of the insurers.

**Conclusion:**

Important methodological limitations and inconsistencies among the studies considered make it extremely difficult, at the moment, to justify policy action taken on the basis of evidence alone. Nonetheless, other empirical and theoretical factors have emerged (for example, the prevalence and impact of the fear of genetic discrimination among patients and research participants, the (un)importance of genetic information for the commercial viability of the private life insurance industry, and the need to develop more equitable schemes of access to life insurance) that should be considered along with the available evidence of genetic discrimination for a more holistic view of the debate.

## Background

The prototypical issue used when discussing the ethical, legal and social issues associated with scientific progress in genetics has been genetic discrimination (GD). Lawyers and ethicists have been quick to point out the risk that uninhibited genetic progress would entice governments and institutions to treat people differently on the basis of their genetic constitution [1]. GD has been defined in many ways, a mark of the influence of divergent sociocultural and scholarly backgrounds. Insurers write of 'rational (actuarial)-irrational discrimination' [2], lawyers write of 'legal-illegal (illicit) discrimination' [3], whereas patients generally adopt a much broader definition encompassing all differential, negative treatments of an individual based on his or her genetic makeup [4]. However defined, widespread GD could potentially result in practices that exclude segments of the population from access to basic social necessities such as healthcare, insurance, housing, reproductive freedom and employment. Mass media has joined the debate, ensuring that the issue of GD is not confined to isolated academic discourse [5].

Among the fields of potential discrimination, one of the most commonly-debated topics has been the use of genetic information by the insurance industry to select applicants and determine insurance premiums. The dual nature of personal insurance, which is partly considered as both a public and private good in most jurisdictions, and the relatively limited amount of public trust in the practices of the private insurance sector might explain some of this attention. Policymakers themselves have entered the arena of debate following substantial pressure from their constituents. In continental Europe, the legislative response has been swift and strong. GD is prohibited by the *Convention on Biomedicine *(1997), the *Charter of Fundamental Rights of the European Union *(2000), and the national legislation of many individual countries [6]. In the United States, the much-discussed *Genetic Information Nondiscrimination Act of 2008 *(*GINA*) (2008) offers protection mainly in the domains of health insurance and employment [7]. In the United Kingdom, the Association of British Insurers and the British government have agreed on a *Concordat and Moratorium on Genetics and Insurance *that significantly restricts the capacity of British insurers to request genetic information from insurance applicants [8]. Australian (2008 amendment to the *Disability Discrimination Act*), Canadian and East Asian policymakers have also been active in this area, although less so than their European counterparts [6,9].

This paper focuses on GD in the field of life insurance. Life insurance facilitates the economic security of the policy holder. It is often described as a quasi-essential social good, a gateway good necessary to have access to important social and economic activities that provide considerable peace of mind to the policyholder [10]. Access to life insurance is far from universal and it must generally be purchased through a contractual agreement with a private insurance company. The majority of life insurance applicants are accepted at a standard rate set by insurance companies. Nevertheless, for the small group of individuals excluded from the common pool, the consequences can be dire [11].

Is the substantial attention given to the question of GD in academic literature, popular media and policymaking circles justified by the empirical evidence currently available? In other words, are the observed concerns and responses based on documented cases of discrimination, anecdotes or other less visible factors? This question prompted us to undertake a study, which to our knowledge is the first attempt to systematically review all available empirical evidence of GD using the life insurance sector as a subject of analysis. This study analyzes actual cases of discrimination, the evidentiary limitations, and the possibility of drawing overarching conclusions from the available evidence.

## Methods

To assess the state of the evidence, we looked for all studies published in the scientific literature documenting the occurrence of GD in the context of life insurance.

### Selection of publications

We developed and applied the following inclusion criteria to identify and select eligible studies: published in English; focused on the collection or capture of information on the occurrence of GD in life insurance whether direct (through patients' or participants' self-report, such as interviews or surveys) or indirect (through professionals such as doctors, genetic counselors and insurers); focused on a primary or follow-up study (multiple publications on the same study were grouped together and treated as a single study). Narrative and systematic reviews were included only if they also presented additional empirical data on the occurrence of GD in the context of life insurance (that is, other than the data contained in the studies reviewed therein).

Eligible studies were excluded if: they did not focus in whole or part on the occurrence of GD in the context of life insurance; they offered insufficient evidence; or they contained serious methodological flaws. Editorial letters with no primary data, comments, opinions, abstracts and unpublished studies were excluded. Studies capturing the fear of GD rather than actual experiences were also excluded.

### Literature search

Searches were conducted from March until May 2012 on PubMed, Google Scholar, Social Science Research Network and Hein Online, using the Boolean operators 'OR' and 'AND' with various permutations of the following keywords: 'genetic discrimination', 'study', 'life insurance', 'survey', 'data', 'empirical evidence' and 'genetic test'.

First, we conducted keyword searches that produced a list including 534 search results. Using the terms 'genetic discrimination' and 'life insurance' combined ('AND'), we obtained a list of 29 publications from the database PubMed. We executed a search on Google Scholar including all the following keywords combined ('AND'): 'genetic discrimination', 'study', 'life insurance', 'survey', 'data' and 'empirical evidence'. This produced 155 search results. Our search on Social Science Research Network using the terms 'genetic discrimination' yielded 100 results. Our last search was conducted on Hein Online using the following combination of terms '("genetic discrimination" AND "life insurance") AND "genetic test" OR "data" OR "empirical evidence" OR "survey"', and generated 250 results.

Second, applying our selection criteria, we reviewed the titles and available abstracts of each of the 534 search results identified through our keyword searches, and retained 29 publications.

Third, we performed a systematic hand search in the references list and bibliography of each of the 29 articles to identify additional relevant publications and assess cross-referencing. This led to 16 more eligible studies. Hence, we uncovered a total of 45 eligible studies relevant to GD in the context of life insurance.

**Figure 1:**
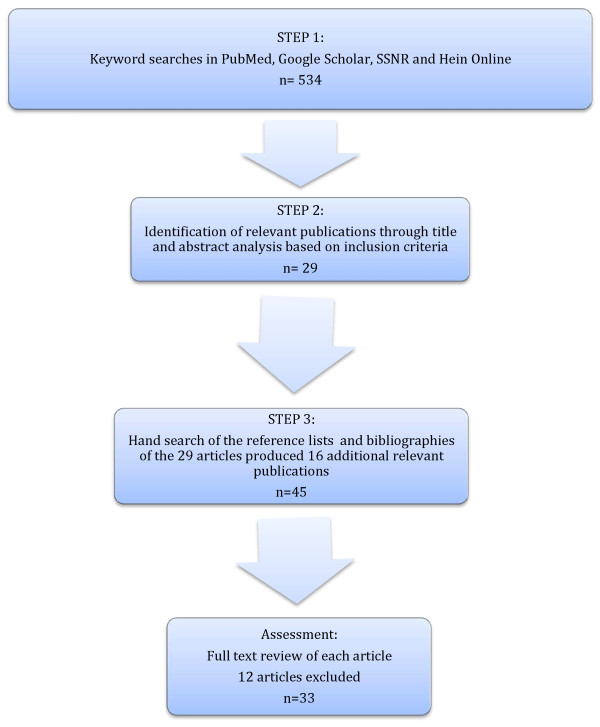
**Methodology for selecting eligible studies**.

Thereafter, eligible studies were each independently assessed for relevance by two researchers (YJ, INF). Applying the selection criteria above, we retained studies documenting experiences of discrimination through both direct and indirect evidence on the occurrence of GD. Based on these criteria, 12 studies were excluded. This final list was also compared with references and resources provided by a recent review dealing with GD in numerous contexts including life insurance [9]. The 33 studies retained represent a systematic overview of the empirical evidence of GD in the field of life insurance. For an overview of the search strategy, see Figure 1.

### Data extraction

The vastly different nature of the available studies suggested that formal statistical analysis and comparison of discrimination cases would be inappropriate. Instead, we analyzed the data through a social science comparative approach that incorporates both quantitative descriptive analysis and qualitative content analysis. Key elements of the selected studies were coded independently according to their relevance to pre-selected themes. We extracted information on study scope (country and context), genetic conditions (whether the study focused on a single or multiple conditions), definition of GD (how GD was construed by the researchers), validation (whether a formal validation process was disclosed by the authors), conclusions (how results were qualified: whether evidence of GD was found, whether policies or laws were required), and number of citations (how often the publication was cited by peers). Two trained researchers (YJ, INF) independently evaluated the results and discrepancies were resolved by consensus. Results obtained on individual themes were qualitatively analyzed and, where appropriate, converted to statistical data (values were rounded according to convention; this process was based on the information presented in Table 1[12-44]).

**Table 1:** Revised studies.

Reference	Date	Scope	Title	Genetic conditions	Study specific to life insurance	Validation process provided	Conclusions on GD evidence^a^	Additional comments/existing policies
[12]	1991	United Kingdom	Cholesterol screening and life assurance.	Familial hypercholesterolemia.	Yes	No	1	
[13]	1992	Canada, United States	Discrimination as a consequence of genetic testing.	A variety of genetic conditions including Huntington's disease, Friedreich ataxia, Charcot-Marie-Tooth, hemochromatosis, phenylketonuria and others.	No	No	1	
[14]	1992	United States	A survey of state insurance commissioners concerning genetic testing and life insurance.	Spina bifida, Huntington's disease, cystic fibrosis, breast cancer, coronary artery disease and sickle cell anemia.	Yes	No	2	
[15]	1993	United States	A survey of medical directors of life insurance companies concerning use of genetic information.	Spina bifida, Huntington's disease, cystic fibrosis, breast cancer, coronary artery disease, and sickle cell anemia.	Yes	Yes	1	
[16]	1994	United States	Genetic discrimination and screening for hemochromatosis.	Hemochromatosis.	No	Yes	2	
[17]	1996	United States	Individual, family, and societal dimensions of genetic discrimination: a case study analysis.	Huntington's disease, phenylketonuria, hemochromatosis, and mucopolysaccharidoses.	No	Yes	2	
[18]	1996	United States	Genetic discrimination: perspectives of consumers.	Conditions were not enumerated but 101 different primary genetic disorders were represented.	No	No	1	
[4]	1998	United Kingdom	Genetic discrimination in life insurance: empirical evidence from a cross sectional survey of genetic support groups in the United Kingdom.	Cystic fibrosis, Huntington's disease, Marfan syndrome, muscular dystrophy, myotonic dystrophy, neurofibromatosis and tuberous sclerosis.	Yes	No	2	*Concordat and moratorium on genetics and insurance*
[19]	1998	United States	Health, life and disability insurance and hereditary non-polyposis colorectal cancer.	Non-polyposis colorectal cancer.	No	No	2	
[20]	1998 to 1999	Canada, United States	"Genetic discrimination": results of a survey of genetics professionals, primary care physicians, patients and public.	Conditions were not enumerated.	No	No	2	
[21]	2000	Europe	Insurance considerations for individuals with a high risk of breast cancer in Europe: some recommendations.	Various conditions, including breast and ovarian cancer, Von Hippel-Lindau syndrome and hereditary non-polyposis colon cancer.	No	No	2	Some of the countries included in the study had adopted laws or policies
[22]	2000	Norway	Health, life and disability insurance and hereditary risk for breast or colorectal cancer.	Breast and colorectal cancer.	No	No	2	*Act of 5 December 2003 No. 100 relating to the application of biotechnology in human medicine*
[23]	2001	Australia	Genetic discrimination in Australia.	Various genetic conditions, including breast and ovarian cancer, inherited bowel cancer (familial adenomatous polyposis), inherited bowel cancer (hereditary non-polyposis colorectal cancer), familial melanoma, rare cancer syndrome, familial early-onset Alzheimer disease, Huntington's disease, rare late-onset neurodegenerative disorders (spinocerebellar ataxia), hemochromatosis, familial high cholesterol (hyperlipidemia), neuromuscular disorders, nervous system disorder (Charcot-Marie-Tooth disease), prion disease (Creutzfeldt-Jakob disease), connective tissue disorder (Marfan syndrome) and myotonic dystrophy.	No	No	1	
[24]	2002	Netherlands	Getting insurance after genetic screening on familial hypercholesterolemia; the need to educate both insurers and the public to increase adherence to national guidelines in the Netherlands.	Familial hypercholesterolemia.	Yes	No	1	*The Act on Medical Examinations *(1998)
[25]	2003	United States	Life insurance and breast cancer risk assessment: adverse selection, genetic testing decisions, and discrimination.	Breast cancer.	Yes	No	3	
[26]	2003	United States	Insurance, employment, and psychosocial consequences of a diagnosis of hereditary hemochromatosis in subjects without end organ damage.	Hemochromatosis.	No	Yes	1	
[27]	2004	United States	Perceptions of genetic discrimination among at-risk relatives of colorectal cancer patients.	Colorectal cancer.	No	No	2	
[28]	2004	United Kingdom	Effect of statin treatment for familial hypercholesterolaemia on life assurance: results of consecutive surveys in 1990 and 2002.	Familial hypercholesterolemia.	Yes	No	3	*Concordat and moratorium on genetics and insurance* Follow-up study to [12]
[29]	2004	United Kingdom	Psychosocial impact of breast/ovarian (BRCA1/2) cancer-predictive genetic testing in a UK multi-centre clinical cohort.	Breast and ovarian cancer.	No	No	1	*Concordat and moratorium on genetics and insurance*
[30]	2007	United Kingdom	Predictive genetic testing for BRCA1/2 in a UK clinical cohort: three-year follow-up.	Breast and ovarian cancer.	No	No	1	*Concordat and moratorium on genetics and insurance* Three- year follow-up study to [29]
[31]	2007	Canada, United States	Genetic screening for iron overload: no evidence of discrimination at 1 year.	Hemochromatosis.	No	Yes	2	
[32-34]	2007 to 2009	Australia	(1) Investigating genetic discrimination in Australia: perceptions and experiences of clinical genetics service clients regarding coercion to test, insurance and employment. (2) Investigating genetic discrimination in Australia: a large-scale survey of clinical genetics clients. (3) Verification of consumers' experiences and perceptions of genetic discrimination and its impact on utilization of genetic testing.	Hereditary hemochromatosis; inherited predisposition to blood clots (hereditary thrombophilia); hereditary breast and ovarian cancers; hereditary bowel cancer (familial adenomatous polyposis and hereditary non-polyposis colon cancer; familial melanoma; rare syndromes such as multiple endocrine neoplasia, Von Hippel-Lindau syndrome; neurodegenerative conditions (spino-cerebellar ataxia; Huntington's disease; early-onset Alzheimer disease; motor neuron disease; prion disease); familial hypercholesterolemia; familial hypertrophic cardiomyopathy; hereditary hypertension; hereditary emphysema (for example, α-1 antitrypsin deficiency); adult polycystic kidney disease and 'other'.	No	Yes	1	All three publications relate to the same study
[35]	2007	Australia	The use of legal remedies in Australia for pursuing allegations of genetic discrimination: findings of an empirical study.	No particular genetic condition was sought after but case result pertained to sickle cell.	No	Yes	3	
[3]	2007	Australia	Investigating genetic discrimination in the Australian life insurance sector: use of genetic test results in underwriting 1999-2003.	No particular genetic condition was sought after but results concerned: hereditary hemochromatosis, Huntington's disease, breast and ovarian cancer, cerebral autosomal dominant arteriopathy with subcortical infarcts and leukoencephalopathy, colorectal cancer, hereditary non-polyposis colorectal cancer, familial adenomatous polyposis, thrombophilia factor V (Leiden) mutation, prothrombin gene mutation, Charcot-Marie-Tooth disease, Marfan syndrome, myotonic dystrophy, multiple endocrine neoplasia, polycystic kidney disease, and spinocerebellar ataxia.	Yes	No	2	Study related to Otlowski et al, 2007 [32-34]
[36]	2008	Canada	Engagement with genetic discrimination: concerns and experiences in the context of Huntington disease.	Huntington's disease.	No	No	1	
[37]	2009	Canada	Perceptions of genetic discrimination among people at risk for Huntington's disease: a cross sectional survey.	Huntington's disease.	No	No	1	
[38]	2009	Austria, Germany, United States	"A slap in the face". An exploratory study of genetic discrimination in Germany.	Huntington's disease.	No	Yes	1	*Austria, Gene Technology Act of 1995* *Germany, Law on Genetic Diagnostic*, 2009
[39]	2009	United States	Survey of unaffected BRCA and mismatch repair (MMR) mutation positive individuals.	BRCA.	No	No	2	
[40]	2010	Netherlands	Obtaining insurance after DNA diagnostics: a survey among hypertrophic cardiomyopathy mutation carriers.	Hypertrophic cardiomyopathy.	No	No	2	*The Act on Medical Examinations *(1998)
[41]	2010	Australia, Canada, United States	Perception, experience, and response to genetic discrimination in Huntington disease: the international RESPOND-HD study.	Huntington's disease.	No	No	2	Australia, *2008 amendment to the Disability Discrimination Act* US, *Genetic Information Nondiscrimination Act of 2008 (GINA)*
[42]	2010	United States	Views of discrimination among individuals confronting genetic disease.	Huntington's Disease, breast cancer, and Alpha-1 antitrypsin deficiency.	No	No	2	US, *Genetic Information Nondiscrimination Act of 2008 (GINA)*
[43]	2012	Canada	Beyond the patient: the broader impact of genetic discrimination among individuals at risk of Huntington disease.	Huntington's disease.	No	No	1	
[44]	2012	Netherlands	Improved access to life insurance after genetic diagnosis of familial hypercholesterolaemia: cross-sectional postal questionnaire study.	Familial hypercholesterolemia.	Yes	No	2	*The Act on Medical Examinations *(1998) Follow-up to [24].

^a^1 = exist and is a concern; 2 = limited, present only marginal concern; 3 = no evidence; GD: genetic discrimination.

## Results and discussion

### Scope and context

Together, the 33 studies represent two decades' worth of research published from 1991 to 2012 (see Table 1). A small peak in the number of studies (eight studies) can be identified between 2006 and 2007, which can be linked to the charged political climate in the US and renewed academic interest that prefaced the adoption of *GINA*. These studies generally gathered evidence through direct sources (that is, individuals commenting on their own experience with insurance companies), but some obtained their data through secondary sources such as insurers, insurance associations, health professionals or patient groups.

**Figure 2:**
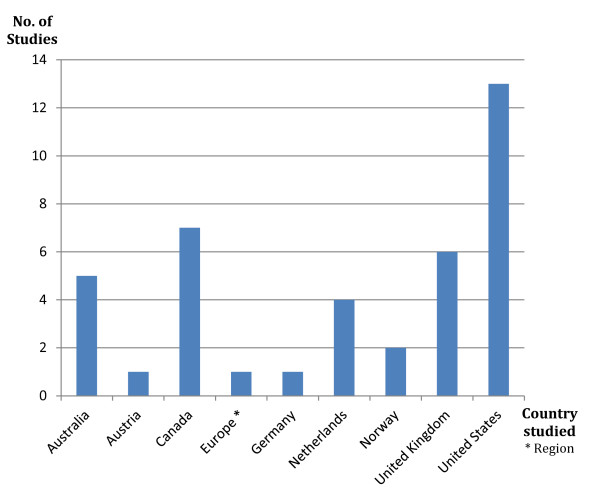
**Studies on genetic discrimination and life insurance by country**.

The majority of studies reviewed (73%) aimed at providing evidence of GD in a variety of fields (for example, employment, immigration, adoption and access to healthcare), including that of life insurance. A minority (27%) focused exclusively on the context of life insurance (see Table 1). The majority of the available evidence (58%) comes from studies involving North American population groups (see Figure 2 and Table 2).

**Table 2:** Distribution of results on the evidence of genetic discrimination.

	**Results by region studied**			**All studies (33)**				
**Conclusions on the evidence of GD**	**Europe** **(11)**	**US and Canada** **(19)**	**Australia** **(5)**	**Studies on single condition** **(n = 19)^a^**	**Studies on multiple conditions** **(n = 14)^a^**	**Validated studies** **(n = 8)^a^**	**Non-validated studies** **(n = 25)^a^**	**Overall (n = 33)**
				
**(1) GD exists and is a concern**	5	8	2	9	5	4	10	14
**(2) GD exists but is rare and exceptional**	5	10	2	8	8	3	13	16
**(3) There was no evidence of GD**	1	1	1	2	1	1	2	3

^a^Out of 33. GD: genetic discrimination.

Surprisingly, given the rather strong European policy response mentioned above, only six studies (18%) provided empirical data on the situation prevailing in continental Europe, and five of these studies were very specific in nature, addressing the context of familial hypercholesterolemia and hypertrophic cardiomyopathy in the Netherlands, hereditary breast and colorectal cancer in Norway, and Huntington's disease in Germany [24,38,40,44].

The substantial number of studies carried out in Canada (seven studies), where no specific laws have been adopted to limit the use of genetic information by life insurers, and the absence of studies in a highly legislated European context, could suggest that the number of GD studies carried out in a given country, as well as the number of GD cases reported, does not necessarily have a strong correlative impact on policymaking. A notable exception to this trend could be Australia, whose Disability Act was amended following the publication of major studies on GD and an extensive report from the Australian Law Reform Commission [45].

### Genetic conditions investigated

The mitigated overall results are not easily interpretable and the task is exacerbated by the serious methodological challenges faced in some of these studies. One of the constraints concerns the range of genetic diseases investigated in the literature. Reviewing the scope of the 33 studies, it is apparent that they only uncovered evidence on a very limited number of highly penetrant, familial, adult-onset, relatively well-known genetic conditions.

**Figure 3:**
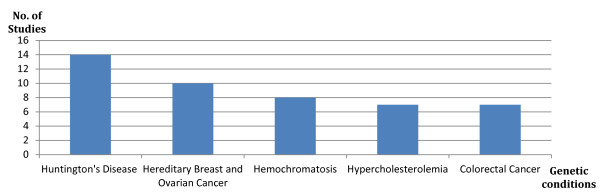
**Most studied genetic conditions in genetic discrimination and life insurance studies**.

**Figure 4:**
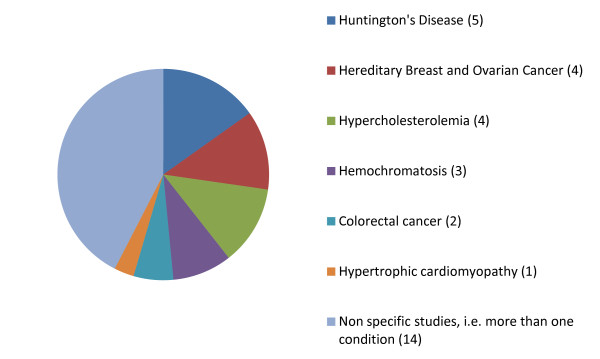
**Proportion of studies that focused on a single disease**.

The majority of the evidence is based on the following five conditions: Huntington's disease, hereditary breast and ovarian cancer, hemochromatosis, familial hypercholesterolemia and hereditary colorectal cancer (see Figure 3). Of the 33 studies reviewed, 19 (58%) specifically focus on one of these five conditions (see Figure 4). Moreover, evidence on genetic discrimination in the context of Huntington's disease is provided in over 14 of the 33 studies, and on hereditary breast and ovarian cancer in more than 10 (see Figure 3).

The high number of studies focusing on a single condition (see Figure 4) makes it particularly difficult to generalize from the results of a systematic comparison of the literature to reach a broad, robust conclusion on GD applicable to the whole research or clinical genetic context. Moreover, research on GD in the fields of personalized medicine and/or pharmacogenomics, infectious diseases and genome-wide association studies remains absent in the literature, thereby resulting in a complete lack of data on GD in the context of emerging 'omics' research. This is particularly concerning given that the amount of genomic information in the typical individual's medical record is likely to increase tremendously in the next few years as whole-genome sequencing costs are reduced and personalized medicine becomes more common in clinical settings [46].

### Definition of genetic discrimination

The authors of the 33 studies all struggled with the meaning of GD (examples of GD definitions are provided in Table 3). Several studies refrained from using the term 'genetic discrimination' in their questionnaire so as to avoid biasing responses. However, the paradoxical consequence of this methodological approach was over-reporting due to the tendency of participants to declare any negative outcome they faced while applying for life insurance as an instance of discrimination [47]. When specifically included in survey questionnaires or data analysis strategies, the definition of GD varied widely (see Table 3), greatly reducing the possibility of meaningful comparison. Indeed, the challenge of defining GD led a study author to conclude that '[t]he notion of finding wholly objective and overt evidence, as opposed to subjective and implicit accounts of discrimination may [n]ot be entirely realistic' [42]. Studies choosing to adopt a broad definition, or no definition at all, tended to report the highest incidence of GD cases [36]. Studies using a legal definition of GD obtained lower results [3]. But, because laws on this topic vary significantly across jurisdictions, these studies are difficult to compare or integrate with one another outside of their national context.

**Table 3:** Examples of definitions of genetic discrimination.

Authors	Reference	Definition of genetic discrimination
Lapham *et al. *1996	[18]	Prejudicial action as perceived by the respondents that resulted from insurers' or employers' knowledge of an individual's genetic condition, carrier status, or presumed carrier status, based on observation, family history, genetic testing, or other means of gathering genetic information.
Apse *et al. *2004	[17]	When people or organizations make unfair decisions about someone *who is currently healthy *based on genetic information (results of genetic testing or family history information).
Taylor *et al.*, 2007	[34]	Differential treatment of a person who has no manifest symptoms of a condition or disorder and which has occurred allegedly on the basis of their genetic characteristics or makeup, either real or assumed.
Bombard *et al. *2009	[37]	Being unfairly prevented from doing something or being treated unfairly (because of family history or genetic test results).
Erwin *et al. *2010	[41]	The denial of rights, privileges, or opportunities or other adverse treatment based solely on genetic information, including family history or genetic test results.

To obtain more robust and comparable results, some studies have used the criteria of 'irrational discrimination' - discrimination that is not based on scientifically validated and actuarially relevant genetic information - as a selection criterion to assess the practice of insurers. However, because negative decisions by life insurers against some of the most genetically at-risk individuals who might have pressing need to obtain life insurance (for example, an asymptomatic patient having tested positive for a monogenic dominant serious condition) would not necessarily constitute irrational GD, use of this criterion could arguably be perceived as unethical. The partly subjective nature of the underwriting process (illustrated by the high variability between the guidelines and questionnaires used by different insurance companies) and our limited knowledge of the genomics of complex diseases further limit the use of the rationality criterion to determine objectively the prevalence of GD in insurance.

The context of Huntington's disease can be used to illustrate the impact of definitional choices on the results of GD studies. Because this disease is a relatively well-known autosomal dominant genetic condition, obtaining a positive test result has serious implications for the future health of an asymptomatic individual. This explains the life insurers' interest in being able to use test results or family history information, regarding this particular disease, for underwriting. This is in turn reflected in the results of GD studies. Studies investigating GD in the context of Huntington's disease and using a broad definition of GD or no definition at all would likely identify a significant number of GD cases (exclusion, higher premiums or conditional acceptance). However, studies using a rationality or legality criteria would generally report a much lower number of discrimination incidents.

### Evidence of genetic discrimination

Around half of the studies reviewed (48%) found that, although GD had some empirical basis, its incidence was rare and it was not a significant source of insurance denials [4,14,16,17,19-22,27,31,39,40,42,44]. A second category, comprising a considerable number of studies (42%), concluded that the existence of GD in life insurance was documented by the evidence they provided and that the situation gave grounds for serious concern. Within this category, Huntington's disease came up often [36-38,43]. Early US studies in this category often advocated the adoption of laws and policies to prohibit access to genetic information by life insurers [17] or the development of a more generous public insurance system that would provide a minimum amount of life insurance to all applicants [16]. Finally, a minority of studies (9%) found no empirical evidence to support the existence of GD in the life insurance context.

It should be mentioned that some, but not all, of the countries covered by these studies, have already adopted laws prohibiting insurers' access to genetic information (for example, the Netherlands, Norway, and Germany) (see Table 1). Authors of the 9% of studies finding no empirical evidence of GD were often of the opinion that the GD problem was more linked to media hype and fear of discrimination than to GD itself. These studies consequently pointed out the importance of educating the public and reassuring the concerns of patients and research participants about GD [22,40].

Among the 19 studies dealing with a single genetic condition (see Table 2), a significant number of studies (47%) concluded that there was sufficient evidence to raise serious concerns about GD. Half of these studies concerned Huntington's disease [36-38,43]. A second important category (42%) found that, while GD existed, it was of rare occurrence [19,31,39,40,44]. Finally, a minority of studies (11%) concluded there was no evidence of GD [25,28].

To highlight the broad trends, it is possible to further group the 33 reviewed studies into two categories: a majority of studies (58%) that believes that GD in the context of life insurance is a negligible issue that does not warrant the substantial societal debate and policy concern generated to date, while a substantial minority (42%) concludes that GD exists and has impacted access to life insurance negatively.

### Validation and methodological limitations

Validating the study results (that is, avoiding biases and concealments as well as ensuring that the data reflect cases of 'real' rather than 'perceived' discrimination) was another significant hurdle. For the purpose of our research, validation was considered to be any additional independent step(s) or method(s) taken by the researcher to confirm the accuracy of reported discrimination events. A majority of the reviewed studies (76%) could not be considered as validated (see Table 1 and 2); in this case it seems more accurate to talk about studies assessing the 'perceived' level of GD rather than objective manifestations of it. Testifying to the importance of the validation process, Wertz found that, '[W]hen asked to give details of their refusals, almost all [participants] described situations that are characteristic of general insurance practice. They were apparently objecting to what they perceived as unfair insurance practices in general, rather than practices specific to genetics.' [20].

Studies of patients were not the only ones in which investigators noted the importance of verifying findings. Otlowski *et al. *observed that insurers surveyed on the topic of GD were likely to under-report unfavorable underwriting decisions [3].

To attempt to reduce the biases associated with non-validated results, several verification techniques have been used over the years. They include follow-up phone calls or in-person interviews to elicit additional information about reported cases of discrimination; review of the participant's medical file (to verify if an unfavorable decision could be due to a pre-existing condition); audit of the documentation or correspondence relating to any discrimination complaint; corroboration of discrimination reports by independent sources (ombudsman or similar administrative instances); case law; and so on. These validation techniques were used, alone or in combination, with various degrees of rigor by researchers and these choices significantly impacted the results of the reviewed studies.

Other study limitations that impacted the results and their compatibility with one another included the sample size and type of people surveyed. Some studies would include individuals already affected by a genetic disease but asymptomatic [16], whereas others would include healthy carriers [30]. Some would include asymptomatic untested individuals with a family history of disease [37], and some would include information on patients that was obtained from indirect sources (family members, genetic counselors or members of a disease support group) [21]. The lack of large-scale studies of well-characterized individuals also made it difficult to extrapolate from the results to objectively estimate the prevalence of GD in the life insurance sector.

Treloar and colleagues have written that, 'conceptualizing, investigating and verifying individual's experience of genetic discrimination constitute a challenging endeavour' [47]. Discrimination surely can take many subtle forms. For example, rather than charging a higher premium or excluding an applicant, an insurance company could decide to process an application more slowly or ignore phone calls and emails in the hope of discouraging pursuit of the application process. In this case, the applicant might not even be aware that she or he has been discriminated against.

Given these serious challenges, it should come as no surprise that the five most influential studies on GD within academia, as measured by Web of Science and Google Scholar citation rates (see Table 4), all suffer from important limitations. For example, the most cited article on GD in life insurance, a precursor 1992 pilot study by Billings *et al.*, used a broad definition of GD and reported 29 responses describing 41 separate incidents of possible discrimination (32 in the field of insurance) [13]. The study undertook an extensive advertising campaign (1,119 letters mailed to genetic professionals, an advertisement in the American Journal of Human Genetics, and similar advertisements published in several patient organization newsletters) to elicit this relatively small number of potential discrimination cases from an under-defined population that included symptomatic respondents. The authors of the study acknowledged the limitations of their work stating that it is not meant to demonstrate the prevalence or the full range of discriminatory practices. Nevertheless, their conclusion that unfair and discriminatory use of genetic data existed and that new laws and sanctions should be considered does not seem to accord with the limited data and exploratory methodology provided in the study. Learning from early experiences and challenges in this field, more recent studies tend to draw more cautious or qualified conclusions and to recognize their own substantial methodological limitations [32].

**Table 4:** Most cited studies on genetic discrimination in life insurance.

Ranking	Date	Authors	Title	Reference	Number of citations
1.	1992	PR Billings, MA Kohn, M de Cuevas, J Beckwith, JS Alper and MR Natowicz	Discrimination as a consequence of genetic testing	[13]	Web of Science: 287 Google Scholar: 436
2.	1996	EV Lapham, C Kozma and JO Weiss	Genetic discrimination: perspectives of consumers	[18]	Web of Science: 181 Google Scholar: 255
3.	1996	LN Geller, JS Alper, PR Billings, CI Barash, J Beckwith and MR Natowicz	Individual, family, and societal dimensions of genetic discrimination: a case study analysis	[17]	Web of Science: N/A Google Scholar: 127
4.	2004	M Watson, C Foster, R Eeles, D Ashley, R Davidson, J Mackay, PJ Morrison, P Hopwood, DGR Evans and Psychosocial Study Collaborators	Psychosocial impact of breast/ovarian (BRCA1/2) cancer-predictive genetic testing in a UK multi-centre clinical cohort	[29]	Web of Science: 60 Google Scholar: 92
5.	1998	L Low, S King and T Wilkie	Genetic discrimination in life insurance: empirical evidence from a cross sectional survey of genetic support groups in the United Kingdom	[4]	Web of Science: 57 Google Scholar: 99

## Conclusions

This systematic review offers evidence that the literature recognizes the existence of incidents of GD in North America, Australia and the UK. We note four key observations. First, the methodology used in most of the studies is not sufficiently robust to clearly establish either the prevalence or impact of discriminatory practices in these regions. Second, the current body of evidence was mostly developed around a very small number of 'classic' genetic conditions. Third, the heterogeneity and small scope of most of the studies prevent formal statistical analysis of the aggregate results. Fourth, the small number of reported cases of GD in some studies could indicate that these incidents of GD took place due to error(s), rather than voluntary or planned choice, of the insurers.

These observations should not be interpreted as dismissing the importance of the significant work that has been accomplished by researchers in this field over the past 20 years. Our review has allowed us to confirm the existence of GD, to identify large areas of evidentiary gaps (for example, discrimination in the context of 'omics' studies) and methodological challenges (defining GD, verification of reported incidents of GD), and to identify promising methodologies to build upon for future studies such as the one used for the Australian Genetic Discrimination Project. In this project, a rich body of evidence was gathered from a variety of sources with special attention given to validation and methodological concerns [3,32,33,47]. This information can be used by the international research community to continue monitoring and documenting experiences of GD and its psychosocial and economic impact on individuals with improved, more streamlined research strategies.

To return to our original question, can the intense debate around GD in the life insurance context that has taken place this past quarter-century be justified on the basis of the available evidence? We must answer in the negative. With the notable exception of studies on Huntington's disease, none of the studies reviewed here (or their combination) brings irrefutable evidence of a systemic problem of GD that would yield a highly negative societal impact. From an ethical and policy standpoint, looking at the evidence alone suggests that targeted policies (in the case of Huntington's disease) and careful monitoring of the situation as it evolves is likely the most adequate course of action.

Nonetheless, other empirical and theoretical factors have emerged that should be considered along with this empirical data. They include the prevalence and impact of the fear of GD in patients and research participants, the importance (or not) of genetic information for the commercial viability of the private life insurance industry, and the need to develop more equitable schemes of access to life insurance. These factors, along with sociocultural and historical elements linked to particular societies (such as early experience with eugenics), would likely offer a better explanation as to why the GD debate became so polarized in popular and academic media. Finally, we wish to highlight that it remains to be determined whether the current GD dilemma is a sign of a broader discomfort with actuarial practices, public policies and access to life insurance at a time when many increasingly view this type of contractual protection as a good of important psychosocial value that is necessary to obtain other important social and commercial goods in post-industrial countries.

## Competing interests

The authors declare that they have no competing interests.

## Authors' contributions

YJ provided the concept of the study and drafted the manuscript. INF participated in the research and assisted with drafting the manuscript. JS provided comments on the concept of the study and the draft of the manuscript. All authors read and approved the final manuscript.

## Pre-publication history

The pre-publication history for this paper can be accessed here:

http://www.biomedcentral.com/1741-7015/11/25/prepub
